# Safety and treatment volumes achieved following new developments of the magnetic resonance-guided focused ultrasound system in the treatment of uterine fibroids: a cohort study

**DOI:** 10.1186/2050-5736-1-20

**Published:** 2013-10-01

**Authors:** Stephen Derek Quinn, John Vedelago, Lesley Regan, Wladyslaw M Gedroyc

**Affiliations:** 1 Department of Obstetrics and Gynecology, St. Mary's Hospital, Imperial College London, South Wharf Road, London, UK; 2 Deparment of Radiology, St. Mary's Hospital, Imperial College London, Praed Street, London, UK

**Keywords:** Uterine fibroids, Magnetic resonance-guided focused ultrasound, Leiomyoma

## Abstract

**Background:**

This research investigates whether modifications to the magnetic resonance-guided focused ultrasound ablation of uterine fibroid (MRgFUS) system used resulted in improved treatment volumes of uterine fibroids, while maintaining safety.

**Methods:**

This study is a prospective cohort analysis of 34 women undergoing the ExAblate 2100 MRgFUS treatment for their uterine fibroids.

**Results:**

The percentage of non-perfused volume (NPV) achieved with the ExAblate 2100 system was 54.92% compared with 50.49 % with the ExAblate 2000 system over the preceding year (*p* = 0.543). The ExAblate 2100 system resulted in a greater NPV in hyper-intense fibroids compared with the ExAblate 200 system (43.20% versus 36.33%, *p* = 0.005). There have been no recorded hospital admissions, no skins burns, and no reported major adverse events since the introduction of this new system.

**Conclusion:**

Overall, the new system has thus far shown an encouraging safety record and an improvement in non-perfused volumes achieved, especially in hyper-intense fibroids.

## Background

Magnetic resonance-guided focused ultrasound (MRgFUS) treatment of uterine fibroids uses MR guidance to direct high-intensity focused ultrasound into a fibroid, resulting in coagulative necrosis within the tissue. This treatment was first performed in 2002 [1] and received approval from the US Food and Drug Administration in 2004. It is known that the signal intensity of the uterine fibroids (UF) at baseline T2-weighted MR imaging and the non-perfused volume (NPV) of the fibroid post-treatment are important determinants of outcome following MRgFUS [2]. Compared to other treatment options for uterine fibroids, focused ultrasound has been shown to carry a very low risk of complications and side effects [1]. At our institution, uterine fibroids have been treated using focused ultrasound since 2003. In January 2011, a new transducer and software system, the ExAblate 2100 (InSightec, Haifa, Israel), replaced the previous system in use, the ExAblate 2000 (InSightec).

The ExAblate 2100 system incorporates several software and hardware upgrades which were not components of the ExAblate 2000 system. This includes a new 3D axis ultrasound transducer, capable of rotation, tilt, and movement in the vertical plane to allow the transducer to be placed much closer to the patient's skin surface than formerly. The system also allows transducers to be shut off in banks which allows for sculpting and steering of the ultrasound beam. This affords the operator enhanced control in avoiding non-target structures and defining the location of energy dissipation posterior to the fibroid. As with the earlier system, focal spot size, location, and angle, in addition to the exact amount of energy deposited, can all be manually controlled using the 2100 system. In real time, MR thermal maps depict the precise location of treated tissue, providing the operator with a high degree of control throughout the procedure. In addition, the introduction of ‘scar tape’ , an adhesive barrier that prevents build-up of heat within the scar tissue, reduces the risk of cutaneous thermal injury for treating patients who have an anterior abdominal wall scar [3]. This tape enables women with low transverse abdominal scars to be treated safely while avoiding the build-up of excess heat within the scar and subsequent burns. This tape was not available to us when we were treating patients with the ExAblate 2000 system; however, this could be used by other units using the earlier system.

The overall effectiveness of MRgFUS has been linked to the overall volume of fibroid tissue treated, as measured by the NPV [4]. The NPV refers to the volume of fibroid tissue identified as non-enhancing following administration of radio-opaque contrast agent.

We aimed to assess the effectiveness of this new technology by comparing the non-perfused volumes achieved with the newer technology to those obtained using the ExAblate 2000 system. We also aimed to compare the safety profile of the two systems.

## Methods

This was a prospective cohort study of women undergoing treatment for uterine fibroids with the ExAblate 2100 MRgFUS system (*n* = 34). The results were compared with a data collected retrospectively from a cohort of women undergoing treatment using the ExAblate 2000 system prior to January 2011 (*n* = 238).

All patients underwent standard pelvic MRI including T1 and T2 images prior to treatment. All MRI images were reviewed by two independent investigators (SQ and JV). The fibroids were graded as hypo-intense or hyper-intense relative to myometrium and rectus abdominus skeletal muscle. The non-perfused volume following both systems was calculated using the same software package utilizing the planimetric method (Report Card Software, GE Healthcare, Milwaukee, USA) using the images from the sagittal T2 images (5-mm slices). Volume calculation between investigators was tested for agreement using the Bland-Altman method [5] and interclass correlation coefficient.

A range of patient demographic data was recorded, including body mass index, symptom severity score, parity, ethnicity, primary symptoms, and previous treatment. Prospective data was collected for those women undergoing the newer ExAblate 2100 system for 1 year post-treatment. All data regarding complications were recorded via review of patients' notes, interview with patients at follow up, telephone and written questionnaires, and direct observations of patients' post-procedures.

The statistical package SPSS version 20.0 (IBM, Armonk, NY, USA) was used to analyze differences between variables in both groups. Continuous data were tested for normal distribution by Shapiro-Wilk test. Normally distributed data were tested using Student's *t* test; where variables were not normally distributed, Mann–Whitney test was performed to investigate differences between these groups. For the categorical data of hyper-intensity, the differences between those undergoing the ExAblate 2100 and 2000 systems were tested using chi-square testing between these two groups.

The NPV achieved between the groups was investigated for the possible effect of signal intensity differences between groups using Mann–Whitney non-parametric testing. Analysis of covariance was used to adjust for confounding effect of different signal intensities of the fibroids treated. Differences in fibroid numbers and signal intensity between those undergoing the ExAblate 2100 and 2000 systems were examined using chi-square test. A *p* value less than 0.05 was taken to indicate a significant difference between groups.

## Results

The MR images and treatment details from 238 women who had undergone treatment using the ExAblate 2000 system and 34 women undergoing treatment using the ExAblate 2100 system were examined. Mean follow-up from ExAblate 2000 patients was 3.81 years (SD 1.98). ExAblate 2100 patients were followed up over the first year post-treatment. The test for agreement of volume calculation between investigators using the Bland-Altman method [5] found a bias of 5.24 ml (SD 16.46) and interclass correlation coefficient of 0.998. This degree of agreement between the two investigators was judged to be acceptable.

The demographics for the patient cohort are described in Table 1, and pre-treatment clinical features for the two groups are compared in Table 2.

**Table 1:** Demographics of subjects undergoing treatment with the ExAblate 2000 and 2100 systems

		**ExAblate 2000 (*n*** **= 238)**	**ExAblate 2100 (*n*** **= 34)**	***p*** **Value**
Age	Mean years (range)	42.2 (25–84)	39.47 (25–52)	0.26^a^
BMI	Mean (SD)	25.1 (4.6)	23.41 (3.79)	0.038^a^
Race	White, *N* (%)	135 (48.0)	14 (41.18)	0.01^b^
	Black, *N* (%)	76 (27.0)	12 (35.30)	0.01^b^
	Asian, *N* (%)	18 (6.4)	8 (23.52)	0.01^b^
	Arabic, *N* (%)	7 (2.5)	0	0.01^b^
	Mixed, *N* (%)	4 (1.4)	0	0.01^b^
Parity	Mean (SD)	0.51 (0.89)	0.38 (0.82)	0.328^c^
Symptom severity score	Mean (SD)	62.64 (17.80)	61.71 (17.86)	0.841^c^
Hyper-intense fibroids	*N* (%)	67 (28.2)	6 (17.6)	0.196^b^
Previous treatment	*N* (%)	43 (15.4)	5 (13.9)	0.88^b^
Number of fibroids	Mean (SD)	6.43 (6.54)	4.18 (3.85)	0.044^c^
Mean uterine volume	ml (SD)	788.12 (408.01)	659.62 (259.91)	0.093^c^

*BMI* body mass index.

^a^Two-tailed *t* test.

^b^Chi-square test.

^c^Mann-Whitney test.

**Table 2:** Comparison between ExAblate 2000 and 2100 treatments

	**ExAblate 2000 system (*n*** **= 238)**	**ExAblate 2100 system (*n*** **= 34)**	***p*** **Value**
Mean volume of fibroids treated, ml (SD)	337.78 (252.75)	305.12 (206.28)	0.04^a^
Mean NPV, ml (SD)	165.41 (136.39)	162.39 (19.28)	0.65^a^
Mean percentage NPV (%)	43.72	54.92	0.007^a^
% NPV in hypo-intense fibroids	46.79	57.43	0.005
% NPV in hyper-intense fibroids	36.33	43.20	0.005^a^

*NPV* non-perfused volume.

^a^Mann-Whitney test.

The percentage NPV achieved with the ExAblate 2100 system was 54.92% (SD 19.28) compared with 43.72% (SD 22.26) with the ExAblate 2000 system over the whole 9 years, a significant difference (*p* = 0.007). When signal intensity of the fibroids was corrected for differences, the %NPV between the two treatments remained statistically significant (*p* = 0.012). Of the women treated by the ExAblate 2100 system, 17.6% had fibroids found to be hyper-intense on T2-weighted MRI images, compared with 28.2% of those undergoing treatment with the ExAblate 2000 system (*p* = 0.137). The mean NPV achieved in women with hyper-intense fibroids using the ExAblate 2100 was 43.20% compared with 57.43% in those women with hypo-intense UF. This compares favorably with the 36.33% NPV achieved in hyper-intense UF treated with the ExAblate 2000 system.

Three of the women treated with the ExAblate 2100 required a second treatment for their uterine fibroids in order to complete the treatment. These second treatments were done at 1, 2, and 7 months following the initial treatment and were deliberately planned as the volume of fibroid tissue to be treated could not be achieved in a single session.

The details of the ExAblate 2000 treatments by year since 2003 are detailed in Table 3. When compared to the NPVs initially achieved with the ExAblate 2000 system, the NPV increased and reached a statistically significant improvement by 2011 with the introduction of the ExAblate 2100 system (*p* = 0.007). When compared to the NPV achieved in recent years (2009 and 2010), the NPVs are greater with the ExAblate 2100 system; however, this did not reach statistical significance (*p* = 0.543).

**Table 3:** ExAblate treatments in 2003–2011

**Year**	**Mean age, years (SD)**	**Mean BMI (SD)**	**Mean SSS (SD)**	**Mean uterine volume, ml (SD)**	**Mean total fibroid volume, ml (SD)**	**Mean %NPV (SD)**	**% HI dominant fibroid**
2003	41.97 (5.44)	24.95 (4.05)	64.79 (17.47)	809.73 (445.48)	333.8 (209.75)	41.22 (25.66)	29.2
2004	43.87 (5.09)	24.11 (3.17)	62.1 (14.05)	734.20 (323.86)	387.0 (198.31)	47.33 (23.21)	29.0
2005	43.67 (8.93)	27.23 (4.14)	78.12 (16.24)	949.51 (383.33)	544.23 (276.52)	33.4 (20.34)	30.8
2006	42.74 (10.20)	25.00 (4.39)	64.1 (19.13)	958.1 (469.46)	495.1 (291.49)	39.4 (24.31)	26.9
2007	42.25 (5.93)	26.10 (5.55)	59.4 (16.93)	828.65 (446.98)	431.7 (289.64)	40.7 (22.04)	32.0
2008	43.15 (6.97)	23.75 (3.08)	64.1 (19.26)	861.90 (469.25)	445.1 (314.95)	44.54 (21.10)	36.4
2009	41.1 (6.00)	25.37 (5.97)	57.9 (19.04)	611.01 (249.98)*^a^	314.3 (195.75)	51.53 (19.63)	25.92
2010	40.4 (7.67)	25.5 (4.00)	65.46 (19.18)	658.38 (296.37)**^a^	319.2 (241.88)	50.49 (18.23)	13.78*****^b^
2011 (ExAblate 2100)	39.47 (6.53)	23.41 (4.25)	61.02 (17.13)	655.06 (257.49)***^a^	305.12 (203.43)	54.78 (19.01)****^a^	17.1

*BMI* body mass index, *SSS* symptom severity score, *NPV* non-perfused volume, *HI* hyper-intensity of treated fibroid.

*p = 0.004.

**p = 0.015.

***p = 0.005.

****p = 0.007.

*****p = 0.049.

^a^Mann-Whitney test.

^b^Chi-square test.

Following the treatment of 34 women using the new ExAblate 2100 system, there have been no recorded hospital admissions, no skins burns, and no reported major adverse events. All women were discharged from the MRgFUS center within 3 h of their treatment, and only one woman required analgesia to take home (diclofenac orally for 3 days). There were no reported urinary tract infections and no persistent neurological problems or other complications. For patients treated with the ExAblate 2000 system, minor complications were experienced by 4% of patients and included urinary tract infection, urinary retention, PV bleeding, and transient buttock pain. One woman experienced PV fibroid expulsion, there was one skin burn requiring a small surgical intervention, and one case of a persistent neuropathy. No emergency hysterectomies were required following MRgFUS therapy in either group.

## Discussion

Magnetic resonance-guided focused ultrasound ablation, through controlled deposition of high acoustic energy, causes thermally induced coagulative necrosis of leiomyoma cells. The procedure is performed using real-time MRI guidance, allowing the operator to precisely control the location, intensity, and size of the focus of energy deposited. Control with MRI also allows the operator to monitor the temperature of the tissue treated. This technique has been in use at our institution since 2003 in the treatment of uterine fibroids; these data describe and reflect the evolution which has occurred in patient selection and non-perfused volumes since the introduction of the technology.

We have found an overall improvement in the NPV achieved since the introduction of MRgFUS, and the mean NPV achieved so far with the ExAblate 2100 system (54.92%) is the highest in our unit. However, when compared with the mean NPV achieved with the previous system the year before (50.49%), this was not found to be a significant overall improvement. It is possible that the year-on-year difference in NPV from 2003 to 2010 is a reflection of the increasing experience with the MRgFUS procedure and treatment apparatus, and that a similar curve of improvement aided by familiarity with the technology and better patient selection will continue to evolve. This increase in experience will inform future research directions in this area. Recent publications have reported a 54% NPV following prolonged experience with the ExAblate 2000 system [4]. A survey of MRgFUS operators found a target percentage NPV of 76% and a reported achieved NPV of 58% [6].

The year-on-year results do suggest that patient selection for MRgFUS has favored women with smaller uteri, smaller overall fibroid bulk, and a tendency to treat women with fewer hyper-intense fibroids. There is a known reduced efficacy of this treatment in patients with hyper-intense fibroids on T2-weighted MR imaging [2]. As such, a tendency towards treating single hypo-intense fibroids, rather than multiple fibroids or single hyper-intense fibroids, has become evident since the introduction of the technique in 2003, while patient demographic data remain largely unchanged between the two groups. The higher non-perfused volumes achieved with the earlier ExAblate 2000 technology in hypo-intense fibroids in recent years would seemingly support the rationale for this shift in patient selection. The initial experience with the ExAblate 2100 system suggests that we are able to achieve greater treatment volumes even in those women with hyper-intense fibroids.

The safety of the newer ExAblate 2100 system has proven to be encouraging, with no adverse events recorded at present. There were no complications experienced using the new system. In particular, there were no skin burns or evidence of neurologic injury, which were the two most significant complications seen (albeit very rarely) using the ExAblate 2000 system. It is perhaps worth noting that the complications experienced using either the ExAblate 2000 or ExAblate 2100 were, when compared to the reported complication profile of hysterectomy or myomectomy, relatively modest [7].

As with the introduction of any new technology, a learning curve aided by experience and familiarity with the system is to be reasonably expected. We would hope that once operators using the new ExAblate 2100 system gain more experience with this system, the trajectory of NPV improvement should show further positive improvement. A more evidence-based approach to patient selection, favoring patients with smaller, less numerous, hypo-intense fibroids may augment this anticipated process of improvement. Longer follow-up of these patients is required to determine if increases in non-perfused volume in these patients translate to higher patient satisfaction and a reduced re-intervention rate.

## Conclusions

Overall, the new ExAblate 2100 system has thus far shown an encouraging safety record and an improvement in non-perfused volumes achieved, especially in hyper-intense fibroids.

## Consent

All participants consented to be part of this study. Approval from the research ethics committee of St. Mary's Hospital was obtained for the women undergoing treatment and follow-up by the ExAblate 2000 system (00/EA/76E) and for the women undergoing treatment by the ExAblate 2100 system (10/H0724/29).

## Abbreviations

MR: Magnetic resonance; MRgFUS: Magnetic resonance-guided focused ultrasound; MRI: Magnetic resonance image; NPV: Non-perfused volume; SD: Standard deviation.

## Competing interests

This work was carried out while Stephen Quinn was working as the clinical research fellow at Imperial College London, and the funding for this post was provided by the company InSightec.

## Authors' contributions

This paper was written by SQ, with collaboration with collection of data and statistics with JV under the direct supervision of WG and LR. All authors read and approved the final manuscript.
